# Phylogeography of Avian influenza A H9N2 in China

**DOI:** 10.1186/1471-2164-15-1110

**Published:** 2014-12-15

**Authors:** Yuan Jin, Dong Yu, Hongguang Ren, Zhiqiu Yin, Zhisong Huang, Mingda Hu, Beiping Li, Wei Zhou, Junjie Yue, Long Liang

**Affiliations:** State Key Laboratory of Pathogen and Biosecurity, Beijing Institute of Biotechnology, Beijing, 100071 China; School of Life Sciences, Anhui University, Anhui, 230601 China

**Keywords:** Phylogeography, H9N2, China, Influenza A virus, H9N2

## Abstract

**Background:**

During the past two decades, avian influenza A H9N2 viruses have spread geographically and ecologically in China. Other than its current role in causing outbreaks in poultry and sporadic human infections by direct transmission, H9N2 virus could also serve as an progenitor for novel human avian influenza viruses including H5N1, H7N9 and H10N8. Hence, H9N2 virus is becoming a notable threat to public health. However, despite multiple lineages and genotypes that were detected by previous studies, the migration dynamics of the H9N2 virus in China is unclear. Increasing such knowledge would help us better prevent and control H9N2 as well as other future potentially threatening viruses from spreading across China. The objectives of this study were to determine the source, migration patterns, and the demography history of avian influenza A H9N2 virus that circulated in China.

**Results:**

Using Bayesian phylogeography framework, we showed that the H9N2 virus in mainland China may have originated from the Hong Kong Special Administrative Region (SAR). Southern China, most likely the Guangdong province acts as the primary epicentre for multiple H9N2 strains spreading across the whole country, and eastern China, most likely the Jiangsu province, acts as an important secondary source to seed outbreaks. Our demography inference suggests that during the long-term migration process, H9N2 evolved into multiple diverse lineages and then experienced a selective sweep, which reduced its genetic diversity. Importantly, such a selective sweep may pose a greater threat to public health because novel strains confer higher fitness advantages than strains being replaced and could generate new viruses through reassortment.

**Conclusion:**

Our analyses indicate that migratory birds, poultry trade and transportation have all contributed to the spreading of the H9N2 virus in China. The ongoing migration and evolution of H9N2, which poses a constant threat to the human population, highlights the need for a more comprehensive surveillance of wild birds and for the enhancement of biosafety for China’s poultry industry.

**Electronic supplementary material:**

The online version of this article (doi:10.1186/1471-2164-15-1110) contains supplementary material, which is available to authorized users.

## Background

In Asia, the first isolation of H9N2 avian influenza virus was in Hong Kong in the mid 1970s. Since then, H9N2 avian influenza viruses have been primarily detected in ducks, at live poultry markets, in Hong Kong [1]. In the mainland of China, the first outbreak of H9N2 chicken influenza occurred in the Guangdong province in 1994 [2]. Subsequently, the virus rapidly spread across many different regions of mainland China and became the most prevalent influenza virus circulating in poultry such as chicken, duck, and quail [3, 4]. While causing sporadic and endemic outbreaks in China, H9N2 viruses have evolved into distinct lineages in land-based poultry. These lineages include the G1-like (represented by Quail/Hong Kong/G1/97) and Ck/Bei-like or Y280-like (represented by Chicken/Beijing/1/94 or Duck/Hong Kong/Y280/97) lineages, which have been predominant in China since the mid-1990s [5, 6]. In addition, the H9N2 virus has expanded its host range to mammalian species. Ck/Bei/94-like viruses were isolated from domestic pigs in Hong Kong in 1998 [7]. H9N2 virus infection in pig farms was also confirmed in Shandong and several other provinces in mainland China [8]. Most importantly, several infectious cases in humans exhibiting mild respiratory disease have been reported since 1997 from Hong Kong and China [9, 10].

Prior phylogenetic analysis has revealed that H9N2 has donated its six internal genes to highly pathogenic avian influenza (HPIV) H5N1 viruses, which caused the Hong Kong H5N1 outbreak in 1997 [11]. Recently, there is increasing evidence that shows that avian H9N2 virus may act as a source for novel human avian influenza viruses. In March, 2013, a novel avian-origin H7N9 influenza virus was identified in eastern China and quickly spread to many provinces and cities, resulting in many human infections with a high case fatality rate [12]. Studies have demonstrated that novel H7N9 viruses derive six internal genes from various avian H9N2 lineages through continuing reassorment [13–15]. On November, 2013, another newly reassorted avian H10N8 virus, which possesses internal gene cassettes recruited from the poultry H9N2 virus, was isolated from a 73 years old woman [16]. Together, these data indicate that avian H9N2 viruses maintain a genetic resource to cause human infections, either by direct transmission or by the generation of novel strains.

The H9N2 avian influenza virus was widely distributed in different regions of China and occasionally jumps hosts and reassorts with other subtypes of influenza virus, posing a severe public health threat [17]. In the past decade, the genetic and antigenic evolution of the H9N2 virus in China is well documented [18, 19]. However, the geographic diffusion of H9N2 in China is not fully understood. Understanding its geographic spread and migration patterns could help us better prepare for epidemics as well as endemics and provide useful guidelines for epidemiological control. In this study, we track the migration of the H9N2 virus over different regions in mainland China and the Hong Kong SAR using a Bayesian phylogeography approach [20]. With a Bayesian phylogeography analysis, we determined the source, migration patterns, and corresponding demography history of the avian influenza A H9N2 virus that circulated in China.

## Results

### Phylogeography reconstruction of the H9N2 virus in China

**Figure 1:**
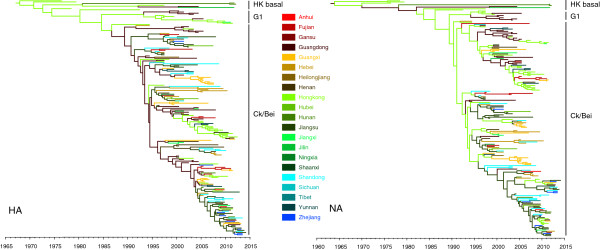
**Maximum clade credibility (MCC) phylogenies for the hemagglutinin (HA) and neuraminidase (NA) genes of avian influenza A H9N2.** The branches are coloured according to the most probable ancestor location of their descendent nodes. The scale bar at the bottom indicates the years before the most recent sampling time (2014).

**Figure 2:**
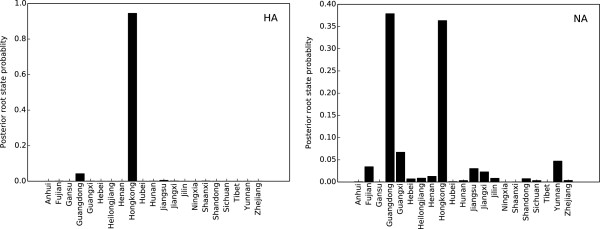
**Posterior root state probability calculated from HA and NA MCC phylogenies.** The histogram shows the posterior probability distributions of root location state of HA and NA.

Phylogeographic studies often focused on the two influenza surface proteins, hemagglutinin (HA) and neuraminidase (NA), as they can change rapidly over a short time period [20–22]. Thus, in this analysis, we used genetic sequences of HA and NA genes to examine the geographic spread pattern for the H9N2 virus in China (including the Hong Kong SAR). Through a Bayesian phylogeography framework, we reconstructed genealogical trees with time-scale and inferred ancestral locations of each branch using sequences’ sampling collection dates and locations. The time-scaled phylogeographic MCC (maximum clade credibility) trees of HA and NA and the root state posterior probability are illustrated in Figure 1 and Figure 2, in which the most probable location of each branch is assigned different colours and the calibrating time-scale is shown on the bottom.

The genealogical tree of HA shows that the early Hong Kong clade and their recent descendants are positioned basal to all G1 and Ck/Bei lineages (Figure 1). The NA gene shows a similar pattern with the exception of some topology differences (Figure 1), which was most likely caused by reassortment [19]. Molecular clock analyses of the HA gene shows that the estimated TMRCA (time of most recent common ancestor) for the root of the H9N2 virus in Hong Kong and mainland China was around Nov 1967 (95% HPD [Apr 1960, Jan 1974]), whereas the TMRCA of NA was around Dec 1963 (95% HPD [Mar 1952, Nov 1973]). The time estimation encompasses the earliest records of H9N2 isolation from a previous report [23].

Spatial reconstruction for HA shows that Hong Kong has the highest root state posterior probability (0.9448). However, as for NA, most probable root state agrees with the Guangdong province (posterior probability 0.3788) instead of Hong Kong (Figure 2). We calculated the Kullback–Leibler (KL) divergence, which estimates the divergence of prior and posterior probabilities of the root state for HA and NA. The NA tree yields lower KL divergence than that of the HA tree (1.3925 vs 2.7747), suggesting a larger uncertainty in the root state estimation. This difference is most likely the result of the deeper root estimation of NA or reassortment in the influenza genome, as these factors increase uncertainty on the root state [20, 24, 25]. Despite the difference, the significantly high posterior root state probability of Hong Kong and larger KL divergence in HA suggests that Hong Kong is most likely the source of the influenza H9N2 migration. Furthermore, we observed that most inferred locations of the trunk nodes in HA and NA trees were the Hong Kong SAR or the Guangdong province, implying that these two places might be responsible for the wide spread of the H9N2 viruses. In addition, either in HA or NA, Hong Kong SAR and Guangdong province share the majority of posterior mass (Figure 2), which highlights both Hong Kong and Guangdong having important roles in H9N2 migration to other locations on mainland China. Thus, our Bayesian phylogeograph analysis indicates that the H9N2 virus in Hong Kong SAR is the source of the influenza H9N2 migration and that neighboring province Guangdong also plays an important role in the virus’s wide geographic spread. Moreover, our time estimation also demonstrates that from approximately 1995 to 2000 (Figure 1), wide migration and vast genetic diversification of the H9N2 virus occurred, during which the virus migrated from Hong Kong and Guangdong to many other interior provinces (Jiangsu, Anhui, Shandong etc.) of China and formed G1 and Ck/Bei lineages (Figure 1).

### Spatial and temporal dynamics of H9N2 virus geographic dispersal

**Figure 3:**
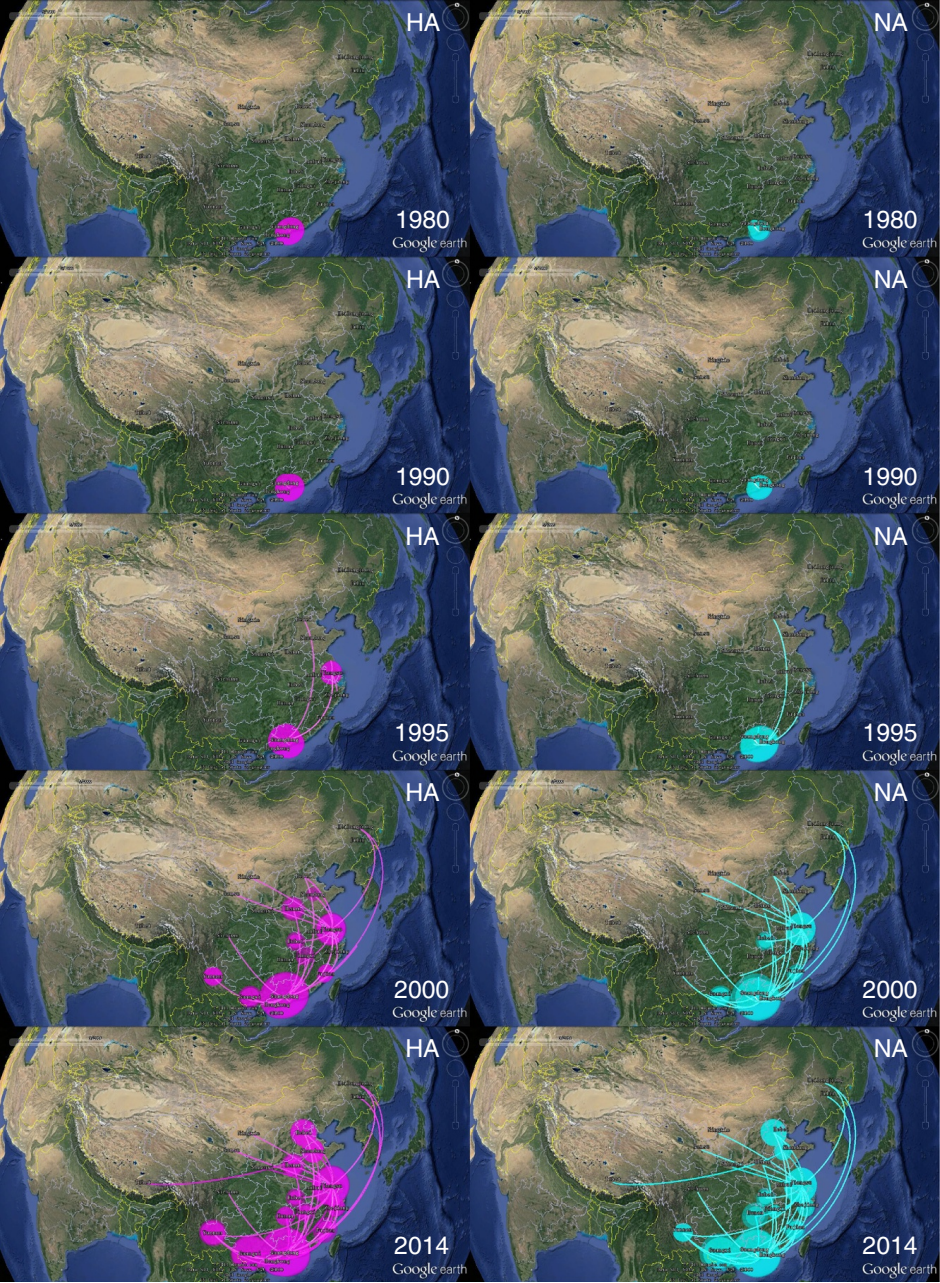
**Temporal dynamics of avian influenza A H9N2 geographic dispersal among different localities of China.** The snapshots show the dispersal pattern of H9N2 virus for 1980, 1990, 1995, 2000 and 2014. Connections between different locations represent branches in the MCC tree along which the relevant location transition occurs. Location circle diameters are proportional to square root of the number of MCC branches maintaining a particular location state at each time point. The white- magenta and white- cyan color gradients show the relative age of transitions for HA and NA, respectively. This map is produced by satellite pictures made available in Google Earth.

To gain insight into the spatial temporal dynamics of the geographic diffusion process of the H9N2 virus in China, we mapped the estimated divergence times and spatial estimates annotated in the HA and NA MCC trees on Google Earth [26]. This mapping enables us to visualise the virus’s geographic spread process over time. The links between different geographic regions represent branches in the MCC tree on which virus migration occur and circle areas reflect the number of branches maintaining a particular location at that time point. The panels in Figure 3 show the temporal dynamics of H9N2 spatial dispersal processes in China.

Before 1980, the H9N2 virus had only accumulated in Hong Kong. Soon after, the earliest dispersal events occurred in which the virus migrated from Hong Kong to Guangdong during 1980–1990. Then, the virus appeared to spread into provinces in East China (Jiangsu and Fujian) and moved as far as North China (Hebei) by 1995. During the next a few years, Guangdong’s role as an epicenter became more apparent. Guangdong showed intensified outbreaks which propagated the virus to the neighbouring provinces of Guangxi and Jiangxi as well as to other locations in China. By 2000, we observed that the viruses had expanded their geography distribution to Central China (Henan and Hubei), Southwest China (Yunnan and Sichuan) and Northwest China (Ningxia and Gansu) and had even reached more remote locations such as Jilin, Heilongjiang in Northeast China. Finally, the H9N2 virus traversed most areas of China and even spread into the Tibet Autonomous Region on the high-elevation, the Qinghai-Tibet Plateau (Figure 3). Despite spreading from the primary epicenter in Southern China, Eastern China, primarily in Jiangsu province, was a secondary epicenter with a large extent of migration links established between Jiangsu and other provinces in East China (Anhui, Shandong, Zhejiang and Fujian), Central China (Hunan and Hubei), North China (Hebei), and Northwest China (Ningxia, Shaanxi and Gansu). The map indicates that the overall migration patterns of the H9N2 virus are from South to North and East to West, although dispersal events also occurred in a reverse direction (Figure 3).

**Figure 4:**
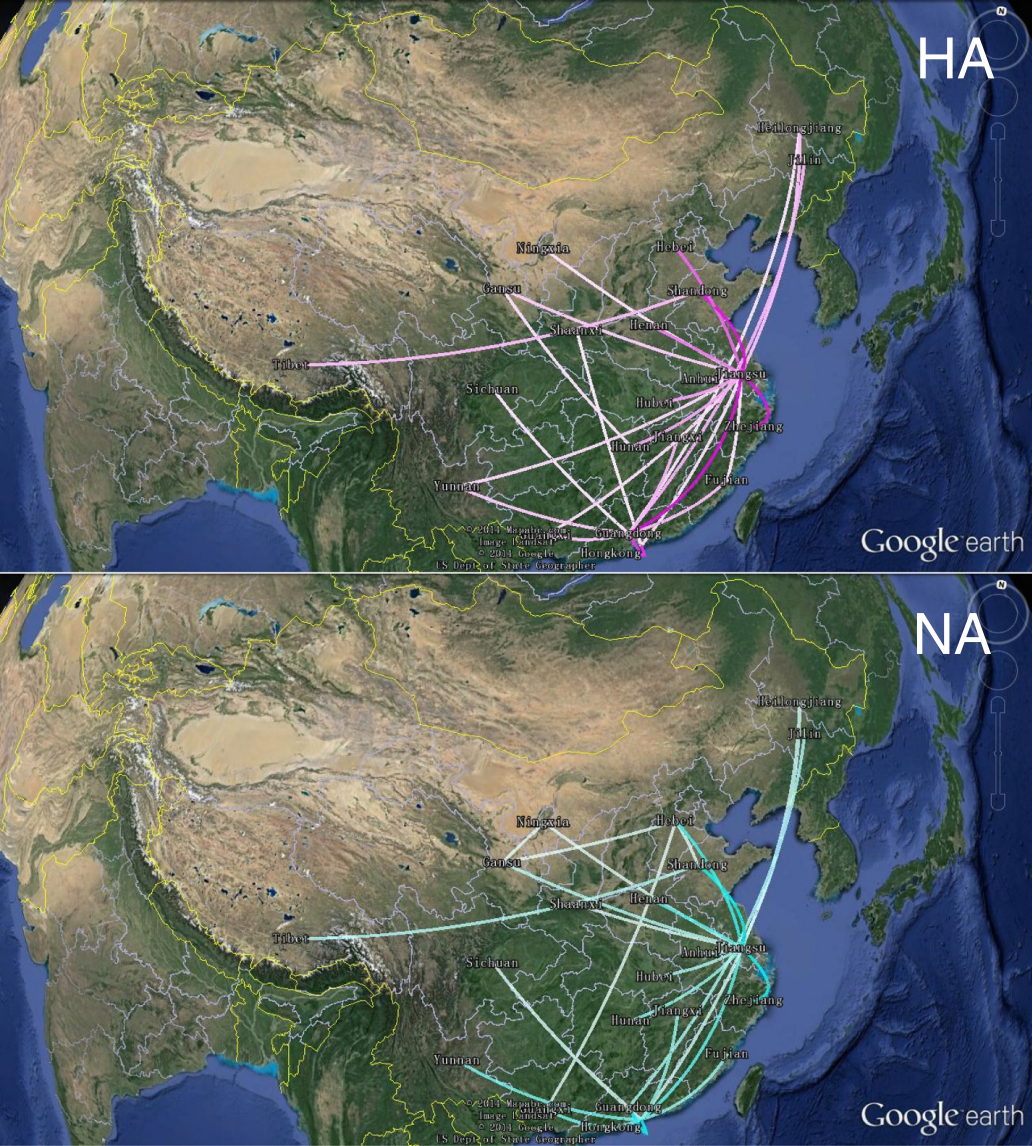
**Significant non-zero dispersion routes for avian influenza H9N2 using a Bayes factor (BF) test with a cut-off of BF = 3.** Lines between different locations indicate transmission routes supported by a BF greater than 3. The colour gradients (HA: from white to magenta; NA: from white to cyan) inform the relative strength of support. This map is produced by satellite pictures made available in Google Earth.

To identify statistically significant transmission routes, we conducted Bayes factor (BF) test for significant non-zero rates between different locations. Using a BF cutoff of three, twenty five significant HA routes were identified, and a majority of the migration links were related to Guangdong and Jiangsu provinces (Figure 4). Guangdong province was closely related to nine locations (Jiangsu, Hong Kong, Jilin, Jiangxi, Fujian, Guangxi, Yunnan, Sichuan, Heilongjiang), and Jiangsu linked with fifteen locations (Guangdong, Shandong, Zhejiang, Hebei, Henan, Hubei, Hunan, Anhui, Heilongjiang, Gansu, Yunnan, Guangxi, Hong Kong, Fujian, Ningxia) (Figure 4). As for NA, most (15 out of 25) of the well-supported rates are consistent with HA, though there are some disparities. Notably, Guangdong only linked to three regions (Hong Kong, Jilin, Sichuan) instead of the nine found in HA and several routes originally connect Guangdong in HA now link Hong Kong (Figure 4). This difference is most likely due to larger uncertainty in the root state estimation for NA (Figure 2). Additionally, similar to HA, Jiangsu province had as many as twelve significant epidemiological links with other locations (Zhejiang, Shandong, Hebei, Henan, Hong Kong, Hunan, Hubei, Gansu, Anhui, Heilongjiang, Ningxia, Shaanxi), and most of the routes were also shared in the HA analysis (Figure 4). Therefore, the Bayes factor analysis for HA and NA shows that Guangdong and Jiangsu provinces were important hotspots for H9N2 migration and confirm the South to North and East to West patterns of H9N2 migration.

### Population dynamics during the H9N2 virus geographic diffusion process

**Figure 5:**
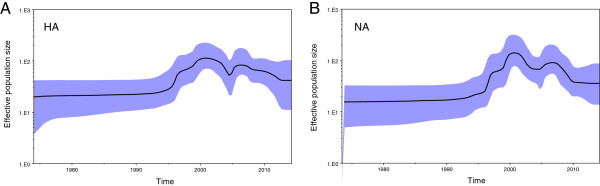
**Bayesian skyline plot of avian influenza A H9N2 in China estimated from HA and NA.** Bayesian skyline plots of the HA **(A)** and NA **(B)** genes show the changes in effective population size (genetic diversity) through time. The thick solid line indicates the median value, and the blue area is the 95% HPD of the genetic diversity estimates.

To examine the changes in genetic diversity during the migration of the H9N2 virus in China, we inferred its demographic history through a Bayesian skyline plots (BSP) coalescent model. A Bayesian skyline coalescent model plots the changing pattern of effective population size through time [27]. Figure 5 shows the BSP reconstruction of the HA and NA genes in which temporal changes of effective population size was plotted.

For HA, the effective population size underwent a nearly constant period from 1970 continuously through the middle 1990s. From approximately 1995, a rapid increase in genetic diversity started and reached its maximum level by 2000. It was during this period that major lineages of China’s H9N2 were generated and became widespread throughout China. After 2000, the effective population size began to decrease, followed by a slight growth. Despite this effect, the reduction of genetic diversity continued, which eventually drove the effective population size to a steady level (Figure 5). Analysis of the BSP for NA genes showed a similar demographic pattern in which no significant changes in the genetic diversity were observed before 1990, followed by a prominent increase from 1995. Following the increase, genetic diversity declined and then stabilised after 2005, despite slight growth.

## Discussion

The above Bayesian phylogeographic analysis suggests that the Hong Kong SAR is the original source of the H9N2 virus circulating in China. In addition, the results indicate that the Guangdong province in southern China is linked to multiple introductions of the H9N2 virus in other geographical areas of mainland China (Figures 1, 2 and 3). While migrating through the entire country, major lineages, such as G1 and Ck/Bei of the H9N2 virus were generated from 1995 to 2000, as demonstrated by TMRCA estimation. This finding is further supported by the BSP analysis in which the effective population size increased rapidly during this time period (Figure 5). The inference is consistent with previous reports and is in accordance with the time in which the H9N2 virus became widespread in poultry throughout China [5, 28].

Our spatial and temporal dynamics for the geographic spread of the H9N2 virus in China revealed more details about its migration patterns. In the map, extensive migration links were established repeatedly from Guangdong, further proving that Guangdong acted as the primary epicentre. This observation supports the idea that southern China is an influenza epicenter because half of the four human influenza pandemics first appeared here; furthermore, several epidemics, including H5N1 and H9N2, occurred here as well [29–31]. Additionally, the diffusion process involves much more than spread from a primary epicenter, and we found that Jiangsu, in eastern China, may have served as a secondary epicentre, seeding tertiary outbreaks in near and remote locations. Hence, southern China, especially Guangdong, perhaps served as the critical source for the spread of the H9N2 virus in China, whereas eastern China, for example, the Jiangsu province, was also an important secondary source to seed outbreaks.

Guangdong and Jiangsu provinces are near the Pearl River Delta Economic Zone and the Yangtze River Delta Economic Zone, respectively, and both are booming markets with advanced transport infrastructures, such as highways. China is the second-largest producer of poultry meat in the world, after the United States, and these two provinces produce the largest amount of poultry in China [32, 33]. Intensive trade and transportation of poultry from the two provinces may largely facilitate the expansion of H9N2’s geographic distribution. In addition, at present, poultry farming in China mainly comes from small individual backyard farms that are free-range and high-density. Additionally, wet markets are important retail channels in China due to the Chinese preference for fresh meat [34]. Such enormous old farms and live poultry markets in China often lack biosafety measures, creating a high risk environment for the spreading of diseases [35–37].

Wild bird migration seemed to impact H9N2 dispersion through the East Asian-Australian flyway. A large majority of the diffusion links, which were established between areas of southern, eastern, northern and central China (Figure 3), are covered by the East Asian-Australian flyway [38]. Every migrating season, in this large region, hundreds of thousands of waterfowl move to southern China in the winter and return to northern and north-eastern China in spring [39, 40]. We found that the initial spread of the H9N2 virus from south to north coincides with birds’ migration pattern (Figure 3). Moreover, we observed several long distance epidemiological links directly connecting Guangdong and Northeast China (Jilin and Heilongjiang) (Figure 3). All of these observations increase the likelihood that migratory wild waterfowl carrying H9N2 may play a prominent role in the dispersal of the virus.

Although multiple lineages of H9N2 occurred as it spread through the whole country via poultry and wild birds, demographic inference suggests that its genetic diversity dropped off after 2000. In addition, from genealogical trees, we observed that the Ck/Bei lineage is the dominant lineages in China, and it continually evolves into antigenically novel strains (Figure 3). Therefore, we believe that the H9N2 virus in China underwent a selective sweep after H9N2’s wide spread and genetic diversification. The ongoing sweep replaced old virus strains with antigenically novel strains, resulting in the reduction of genetic diversity. Importantly, such a selective sweep may pose a great threat to public health because novel strains confer higher fitness advantages than the strains they replace and could generate new virus through reassortment; for example, the A/brambling/Beijing/16/2012 like H9N2 virus, which evolved recently, generated the novel avian H7N9 virus in China [41].

Complex migration dynamics, selective sweep, and co-circulation with other types of avian influenza viruses have made H9N2 in China a notable threat to public health [17, 42]. Understanding its diffusion process and migration patterns is a crucial step for developing effective prevention and control strategies for not only epidemic but also pandemic spreads. Current data shows H9N2 mainly circulates in poultry; therefore, enhancing the biosafety of China’s poultry industry would largely halt the spread of H9N2 as well as future potentially threatening viruses. As large areas of China are covered by migratory flyways, the contribution of wild birds in spreading viruses cannot be ignored; more comprehensive surveillances of H9N2 in wild birds needs to be conducted. In the future, we will continue to gain new insights into the viral migration dynamics and evolutionary ecology in China.

## Conclusions

In this work, Bayesian phylogeography framework was applied to determine the source, migration patterns, and corresponding demography history of the avian influenza A H9N2 virus that circulated in China. Our results suggest that H9N2 in China originated from Hong Kong and subsequently spread to the Guangdong province and other regions. Southern China acts as the primary epicenter for multiple H9N2 strains migrating throughout the country, while eastern China acts as an important secondary source to seed outbreaks. Our demography inference suggests that during the long-term migration process, H9N2 evolved into multiple diverse lineages and then underwent a selective sweep, which reduced its genetic diversity. Our analyses indicate that migratory birds, poultry trade, and transportation may all have contributed to spreading the H9N2 virus in China.

## Methods

### Sequence data preparation and alignment

All hemagglutinin and neuraminidase gene sequences of H9N2 viruses were downloaded from the NCBI Influenza Virus Resource [43]. To reduce the number of sequences, first, HA sequences less than 95% full length were removed, and the resulting HA sequences were clustered using CD-HIT v4.6 [44] with a threshold level of 0.95. Next, we sampled sequences from each cluster and retained strains whose corresponding neuraminidase sequences were also available and took one strain per year, per location, and per host. Thus, our final dataset consisted of 209 H9N2 isolates for which hemagglutinin and neuraminidase sequences were both available. The coding region of the final HA and NA sequences were aligned using MAFFT v7.058 [45] and then were inspected manually according to the amino acid sequences using Mega v5.05 [46].

### Bayesian phylogeography reconstruction

We inferred time-scaled phylogenies of HA and NA by Bayesian Markov Chain Monte Carlo (MCMC) sampling using BEAST v1.8.0 [47]. We used the SRD06 codon position model and the uncorrelated log-normal relaxed clock model under a Bayesian skyline coalescent tree prior in the MCMC simulations [27, 48, 49]. We used the Bayesian sky line plot with a Piecewise constant model to elucidate the population dynamics of H9N2 viruses.

To infer ancestor location and migration events, we grouped the 209 H9N2 isolates into 21 localities including the Hong Kong SAR of China and the Chinese provinces of Anhui, Fujian, Gansu, Guangdong, Guangxi, Hebei, Henan, Heilongjiang, Hubei, Hunan, Jiangsu, Jiangxi, Jilin, Ningxia, Shaanxi, Sichuan, Shandong, Tibet, Yunnan, and Zhejiang. The Chinese municipalities of Beijing and Shanghai were grouped to Hebei and Jiangsu provinces, respectively as they lie in close proximity to these two provinces. Then, the spatial location reconstruction and viral migration were estimated using the discrete Bayesian phylogeographic method implemented in BEAST v1.8.0, which utilised a continuous time Markov Chain (CTMC) over discrete sampling locations, and applied a Bayesian stochastic search variable selection (BSSVS) model [20].

For each data set (HA and NA), we performed four independent runs for 50 million generations with sampling every 5000 steps. Convergence and effective sampling size (ESS) of estimates were assessed by visual inspection using Tracer v1.6 [50]. Multiple chains were then combined after a 10% burn-in using LogCombiner v1.8.0 included in the BEAST package. The maximum clade credibility (MCC) trees with temporal and spatial annotation were summarised with a 10% burn-in removed using TreeAnnotator v1.8.0 in the BEAST package and presentation figures were generated with FigTree v1.4.2 [51].

To provide statistical power for the phylogeographic model, we used the Kullback–Leibler (KL) divergence, which measures the deviation between the posterior and prior probabilities of the root state. To calculate KL divergence, we employed a fixed prior of 1/K, where K is the number of unique states, and with the posterior estimates in Figures 2. Small KL values indicated that the phylogeographic models were not able to produce root state posteriors with strong statistical power [20].

We also conducted Bayes factor (BF) tests to provide statistical support for transmission routes between different geographic locations using SPREAD v1.0.6 with a BF cutoff of three [52]. The Bayes factor (BF) values represent the difference between the posterior and prior probabilities that the rates between two locations are non-zero. Thus, routes with high BF have large odds that a migration exists between two locations.

### Visualizing phylogeographic diffusion

To animate viral dispersal over the time, we converted annotated MCC trees into a keyhole markup language (KML) file using SPREAD v1.0.6 [52], which can be visualised via Google Earth [26]. Example KML files showing avian influenza H9N2 migration processes inferred from HA and NA genes can be found in the Additional file 1 and Additional file 2.

### Availability of supporting data

All gene sequences of H9N2 viruses used in this study were downloaded from NCBI Influenza Virus Resource. Accession numbers of hemagglutinin and neuraminidase gene sequences can be found in additional file 3.

## Electronic supplementary material

Additional file 1: **KML file for H9N2 migration among regions of China over time as inferred from the HA gene.** (ZIP 494 KB)

Additional file 2: **KML file for H9N2 migration among regions of China over time as inferred from the NA gene.** (ZIP 494 KB)

Additional file 3: **Accession numbers of hemagglutinin and neuraminidase gene sequences used in this study.** (XLS 36 KB)
